# Synapse diversity and synaptome architecture in human genetic disorders

**DOI:** 10.1093/hmg/ddz178

**Published:** 2019-07-26

**Authors:** Seth G N Grant

**Affiliations:** Centre for Clinical Brain Science, Edinburgh University, Edinburgh, UK

## Abstract

Over 130 brain diseases are caused by mutations that disrupt genes encoding the proteome of excitatory synapses. These include neurological and psychiatric disorders with early and late onset such as autism, schizophrenia and depression and many other rarer conditions. The proteome of synapses is highly complex with over 1000 conserved proteins which are differentially expressed generating a vast, potentially unlimited, number of synapse types. The diversity of synapses and their location in the brain are described by the synaptome. A recent study has mapped the synaptome across the mouse brain, revealing that synapse diversity is distributed into an anatomical architecture observed at scales from individual dendrites to the whole systems level. The synaptome architecture is built from the hierarchical expression and assembly of proteins into complexes and supercomplexes which are distributed into different synapses. Mutations in synapse proteins change the synaptome architecture leading to behavioral phenotypes. Mutations in the mechanisms regulating the hierarchical assembly of the synaptome, including transcription and proteostasis, may also change synapse diversity and synaptome architecture. The logic of synaptome hierarchical assembly provides a mechanistic framework that explains how diverse genetic disorders can converge on synapses in different brain circuits to produce behavioral phenotypes.

## Synapse proteome complexity and genetic disorders

The brain is the most anatomically complex organ and synapses are the hallmark of this complexity—they are found in vast numbers and their proteome is comprised of thousands of proteins. The discovery in 2000 that the synapse proteome is highly complex (1, 2) transformed concepts of synapse molecular function and has had a major impact on uncovering the role of synapses in disease. The complexity of the synapse proteome became apparent when proteomic mass spectrometry was used to characterize N-methyl-D-aspartate (NMDA) receptors and Membrane Associated Guanylate Kinase (MAGUK) proteins purified from the mouse brain. This revealed that 77 proteins assembled into large multiprotein complexes, and this increased the number of known proteins by 10-fold (1, 2). This discovery opened the possibility that there would be many more proteins in the postsynaptic proteome, which was confirmed by numerous studies (3–10). It is now widely accepted that there is over 1000 highly conserved proteins in the postsynaptic proteome of vertebrate excitatory synapses and several thousand proteins in the overall synapse proteome.

The first clue that the synapse proteome could be the target of many genetic disorders came from the finding that mutations in three of the 77 proteins caused intellectual disability and that mutations in 15 of the 77 genes caused learning impairments in mice (1). Since then, the combination of synapse proteomic and human genetic studies has progressively added to the list of synapse proteins involved with human genetic diseases. There have also been many more studies showing that mice carrying mutations in synaptic proteins show behavioral abnormalities. Characterization of the postsynaptic proteome purified from human brain tissue in 2011 revealed that over 130 brain diseases arise from mutations in hundreds of genes encoding proteins in the postsynaptic proteome of excitatory synapses (5). These diseases include common and rare neurological, psychiatric, neurodevelopmental and neurodegenerative disorders of monogenic and polygenic origin. As human genome sequencing is applied to more brain disorders, the number of genetic variants targeting synapse proteins continues to increase, and this set of proteins appears to be responsible for more brain diseases than any other set of brain proteins.

## Synapses are highly complex and sophisticated signaling machines

The recognition that the synapse proteome is highly complex has required a shift in the basic concepts of synapse physiological function. Before 2000, neurophysiologists had focused a great deal of attention on the concept that the major role of the synapse is to maintain stable synaptic transmission between nerve cells and that changing the stable strength is the primary behavioral function of synapses. With this concept in mind, investigations of molecular mechanisms sought to identify synaptic proteins that would subserve these roles. John Lisman(11) proposed that a mere handful of proteins (the subunits of three protein complexes: the NMDA and α-amino-3-hydroxy-5-methyl-4-isoxazolepropionic acid (AMPA) receptors and the serine-threonine kinase CamKII) in mammalian excitatory synapses would be necessary and sufficient for synaptic transmission and the plasticity underlying learning. However, proteomics showed that these proteins represent fewer than 1% of all proteins in the postsynaptic proteome. Furthermore, genetic studies of many of the other 99% of postsynaptic proteins show that these proteins control synapse stable strength too, as well as the dynamic synapse strength and many different innate and learned behaviors (12, 13). Moreover, the model that stable synapse strength is the core mechanism of learning has been challenged by many genetic and pharmacological dissociations between the synaptic physiology and learning behavior. Thus, the emerging view is that innate and learned behaviors are controlled by the diverse sets of proteins in the synapse and they act in highly integrated and complex molecular networks (14–16). The output of this protein network is the modulation of a plethora of cellular mechanisms ranging from instantaneous control of synaptic strength to regulation of metabolic, proteostatic and transcriptomic cellular mechanisms.

Understanding how the signaling functions of synaptic proteins are integrated requires an understanding of the physical structure and organization of the proteins. Eukaryotic proteins are rarely found as monomers, and almost all are assembled with binding partners into multiprotein complexes (17, 18). A survey of over 60 synaptic proteins found that all were assembled into a hierarchy of multiprotein complexes and supercomplexes (complexes of complexes) (19). The close physical location of proteins and their domains within these supramolecular assemblies confers their integrative functions and sophisticated signaling properties. Disruption of these signaling complexes, as evidenced by mutations in the scaffold protein PSD95 and its interacting proteins, causes behavioral abnormalities and interferes with the ability of synapses to respond to patterns of nerve cell activity (12, 13, 20, 21). This integrative function of multiprotein complexes can explain why mutations in the cognate genes converge to produce similar phenotypes. For example, in mice and humans, there is abundant evidence that PSD95 supercomplexes (also known as MAGUK Associated Signaling Complexes (MASC)) are targets of many human disease genes that cause cognitive impairments including schizophrenia (15, 22–26).

## From synapse proteome complexity to synapse diversity and the synaptome

It has long been known from physiological, pharmacological and anatomical studies that there are different synapse types. For example, in the mammalian central nervous system, the major synapse types can be functionally cataloged into excitatory and inhibitory synapses which use the neurotransmitters glutamate and gamma-aminobutyric acid (GABA), respectively. However, with the advent of gene cloning and molecular labeling methods, it became apparent that this classification was overly simplistic and could not describe the diversity of synapses. There are many subtypes of glutamate and GABA receptor subunits, and combinations of these are differentially expressed in different synapses and confer different physiological properties. When we consider that synapses are built from more than 1000 proteins in many different structural classes and they too are expressed in different combinations, then there is a potentially vast, if not unlimited, number of synapse types. Not only combinatorial usage of proteins can generate synapse diversity but also differential splicing and posttranslational modifications (14, 27). For example, alternatively spliced neurexin isoforms can potentially produce many thousands of different proteins from a single gene, and triggering of neurotransmitter receptors can induce posttranslational changes in hundreds of proteins (14, 27).

Synapse diversity is now beginning to be studied with modern molecular techniques (28, 29) but remains poorly understood for at least two reasons. First, there is a need to develop a conceptual framework and nomenclature to describe the diversity (28, 30). Second, there needs to be tools to characterize the diversity at the scale of the whole brain and not just small samples (31). As a step toward the first issue, the term synaptome was coined to describe the set of synapses in the brain (31, 32). Much as the genome describes the location and features of each gene, the synaptome describes the location and features of each synapse. Just as there has been a set of terms to describe gene structure and genome architecture, there is a need to develop a language to describe the synaptome.

**Figure 1:**
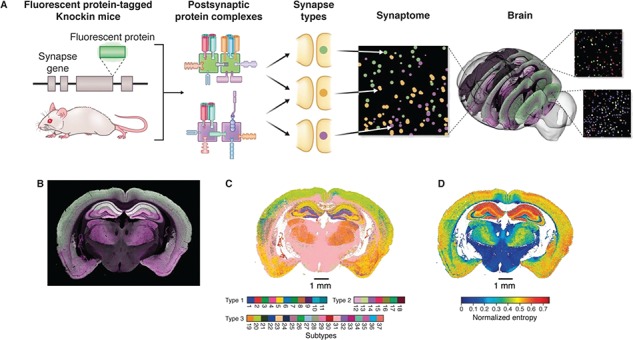
Synaptome mapping in mouse. Genes expressing synapse proteins are tagged in mice by fusing a genetically encoded fluorescent protein onto the C-terminus of postsynaptic scaffold proteins that assemble postsynaptic signaling complexes (PSD95, green; SAP102, magenta). These complexes are distributed into different synapse types that can be visualized with confocal microscopy. The synaptome map is built by quantification of synapse types from regions of the mouse brain. Example image of a coronal mouse brain section showing the differential distribution of PSD95 (green) and SAP102 (magenta). A synaptome map of a coronal section showing the dominant or major subtype from 37 subtypes in different regions. A synaptome map showing the extent of synapse diversity in different regions of the mouse brain. Figures adapted from Zhu *et al.*, 2018 (31).

The first whole brain scale synaptome was recently reported (31). The protein composition and morphological features of ~1 billion individual synapses across all regions of the mouse brain was used to create unbiased synapse catalogs describing synapse diversity and synaptome maps showing the location of different synapse types (Fig. 1). Using high-speed spinning disc confocal microscopy, the amount of two proteins (PSD95 and SAP102) found in the postsynaptic terminal of excitatory synapses together with synapse size and shape parameters was quantified. These two proteins, which were genetically labeled in mice by fusing fluorescent proteins to the C-terminus of the endogenous protein, are required for the assembly of two distinct multiprotein complexes, and thus, the imaging reveals how supramolecular complexes are the building blocks for synapse diversity and synaptome architecture (Fig. 1).

To characterize synapse diversity from the brain-wide data set, a classification scheme that defines synapse types based on the molecular composition of the synapse as the primary feature and the morphology of synapses as a secondary feature was devised (31). Type 1 synapses expressed PSD95, type 2 expressed SAP102 and type 3 expressed both proteins (Figs 1A–C). Addition of morphological parameters enabled each of these types to be further divided into a total of 37 subtypes. Strikingly, each type and subtype showed a unique anatomical expression pattern across the brain. Each region of the brain could be characterized by a particular composition of synapse types and subtypes. Regions of the neocortex and hippocampus showed highest synapse diversity, and basal structures, such as brainstem, showed lowest diversity (Fig. 1D). To facilitate access to the data and visualization of synapses across the brain, a set of maps was compiled into the Mouse Synaptome Atlas resource (http://synaptome.genes2cognition.org) and a versatile viewer called the Synaptome Explorer was developed (31).

The spatial distribution of synapse types and subtypes was shown to be relevant to the connectivity of circuits and behavioral functions. For example, different long-range inputs to the thalamus employed synapses with proteins composed of different combinations of proteins. At the global systems level of the brain, there was a network topology of the synapse composition of different brain regions that correlated with the topology of the functional connectivity of those regions measured with resting state functional magnetic resonance imaging (fMRI). This indicates that the synaptome molecular architecture is relevant to the large-scale electrophysiological network properties of the brain.

**Figure 2:**
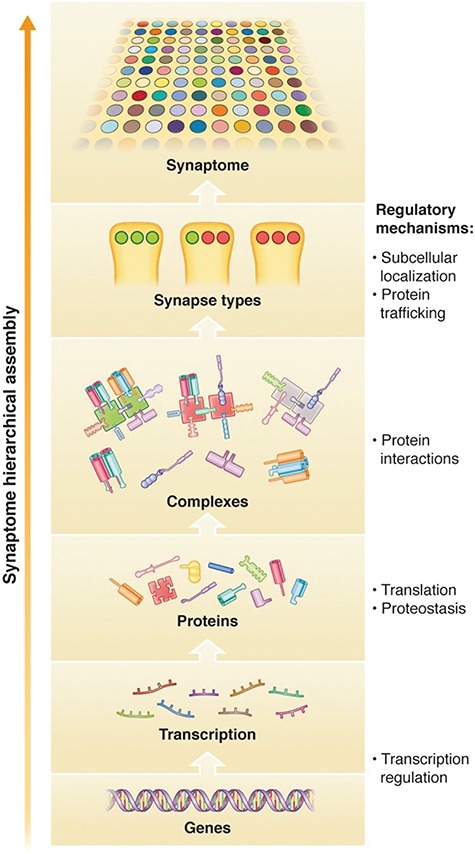
Synaptome hierarchical assembly. The diversity of synapse types and their spatial distribution in the synaptome arise from a hierarchical regulatory mechanism controlling gene and protein expression, assembly of proteins into complexes and supercomplexes and distribution of these supramolecular assemblies into synapses. Mutations acting on regulatory mechanisms at all levels of the hierarchy could influence synapse diversity and synaptome architecture.

The study of synapse diversity arising from only two postsynaptic proteins reveal combinatorial principles that extend to any number of other synapse proteins. Each protein had a unique synaptome map. In other words, each protein was localized into a unique subset of the total number of synapses. As a result, protein combinations generate synapses containing either or both proteins. The number of synapse types arising from *n* different proteins is described as *N*_types_ = 2^*n*-1^ (where *n* = protein number), and 50 proteins (<5% of the synapse proteome) could potentially generate more types than there are synapses in the human brain (5 × 10^14^). With the addition of size and shape parameters, the number of synapse subtypes expands exponentially to a number far beyond the largest and most complex brain of any animal. While there is no doubt that there is vast synapse diversity conferred by molecular combinatorial mechanisms, there are in fact constraints that limit the combinations and diversity. As described above, the synapse proteome of individual synapses is not a soup of promiscuously expressed individual proteins but is a structured assembly of protein complexes and supercomplexes that are built from combinations of proteins, and these supramolecular structures have constraints that limit and define their protein composition (Fig. 2). Considering that synapses are composed of combinations of complexes (and supercomplexes) and these in turn are composed of combinations of proteins, then the impact of a given mutation on a subset of synapses will be determined by the rules of assembly of this molecular hierarchy (Fig. 2).

## Synaptome modification in genetic disorders

Synapse diversity and synaptome architecture have important implications for understanding the mechanism of genetic disorders and where they exert their effects in the brain. In the following section, I will present evidence that suggests that most, and perhaps all, brain diseases will manifest with changes in synaptome architecture and that different diseases will target specific subsets of synapse types. Mutations can act through at least four different mechanisms to change the synaptome:

### Mechanism 1: mutations target subsets of vulnerable synapses

**Figure 3:**
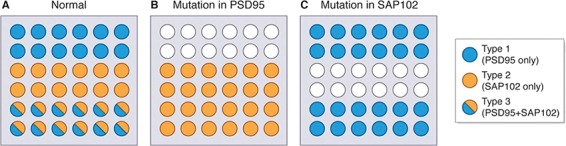
Mutations reprogram synaptome architecture. Model of a normal synaptome comprised of 36 synapses made of three types assembled from two proteins (PSD95 and SAP102). Type 1 synapses express only PSD95, type 2 expresses only SAP102 and type 3 a mixture of both proteins. A mutation that knocks out PSD95 changes the synaptome architecture by abolishing type 1 synapses (empty circles in top two rows) and convert the type 3 synapses to type 2 synapses. A mutation that knocks out SAP102 does not affect type 1 synapses, but abolishes type 2 synapses and converts type 3 synapses into type 2 synapses.

As exemplified by the three synapse types that arise from the combinatorial expression of two synapse proteins, a mutation that results in a change in one protein will affect a subset of synapses (Figs 3A, B). Thus, to understand which synapses (and circuits) are affected by a disease gene, it will be necessary to create a synaptome map of its cognate protein. The subset of synapses within this synaptome map can be considered to be the “genetically vulnerable synapses” and those that are unaffected as the resilient synapses. The versatile synaptome mapping pipeline SynMap is well suited and scalable for creating these maps (31).

### Mechanism 2: mutations induce synaptome reprogramming

Synaptome reprogramming is a fascinating and potentially important regulatory mechanism in disease (31). We found that mutations in PSD93 and SAP102 (PSD93 knockout mice, which are a model of schizophrenia and SAP102 knockouts which are a model of X-linked intellectual disability) changed the synaptome map of PSD95 (Fig. 3C). Thus, a mutation in one synapse protein changes the synaptome map of another synapse protein. To understand the mechanisms involved, we reasoned that because PSD93 is a component of PSD95 supercomplexes then the mutation could change the supercomplex localization and the PSD95 synaptome map. This suggests that mutations in other PSD95 interacting proteins could also change the PSD95 synaptome. However, this mechanism would not apply to SAP102 because it is in physically distinct complexes to those housing PSD95. This suggests that a mutation in a different complex could also change the PSD95 synaptome. Together, these observations suggest that a mutation in any synapse protein could change the synaptome map of PSD95 through synaptome reprogramming. Thus, when we consider how a mutation in a gene could generate a synaptome phenotype, we need to consider the role of mechanisms 1 and 2. At a practical level, it means that, in addition to mapping the synaptome of the mutant protein, it will be important to map the synaptome of other synapse proteins. In this model, we are considering that the connectome anatomy has not changed and it is the synapse proteins that are different. To fully dissect the role of the mutation on the connectome and the synaptome, in future studies it will be useful to use conditional knockout approaches and measurements of dendritic and axonal anatomy.

### Mechanism 3: lifespan temporal synaptome architecture and phenotype penetrance

One of the most puzzling features of some germline mutations is that their phenotypes manifest at late ages and in particular regions of the brain despite the gene being widely and continuously expressed (33). A parsimonious explanation for this phenomenon is that the “molecular context” of the mutation changes with age and brain region, and as a result, the penetrance of the mutation is affected. In the context of synaptic disorders, a change in the synapse diversity with age and brain region might account for a genetic disorder targeting a particular brain circuit at a particular age. Toward this possibility, we have been mapping the synaptome of the mouse brain across the lifespan and find that there are marked changes in the synaptome at different ages (M. Cizeron, Z. Qiu, E. Fransén, S.G.N. Grant, personal communication). It is very likely that the two mechanisms described above will vary with age and brain region, and studying this in the context of genetic disorders may show why some genetic diseases exert their phenotypes later in life.

### Mechanism 4: disruption to the molecular hierarchy that assembles the synaptome

The synaptome is built from a hierarchy of molecular mechanisms from the transcriptome (e.g. the cell-type specific transcriptome), protein turnover (e.g. translation, proteostasis), mechanisms of assembly of complexes into supercomplexes and trafficking of these assemblies into different synapses (Fig. 2) (31, 34, 35). In the discussion above, we have considered the impact of genetic disorders that directly target the synapse proteome and the assembly of complexes and supercomplexes into the synaptome. However, in the broader context of this hierarchical assembly model, there will be mutations that interfere with mechanisms at all levels and these will be expected to impact on synapse diversity and synaptome architecture. Although there is much research to be conducted toward understanding how basic cell biological mechanisms control the synaptome architecture of the whole brain, focused studies have shown that mice carrying a mutation in Fmr1, an RNA binding protein involved with autism, cause changes in distinct subpopulations of synapses in mouse neocortex (36). It is likely that synaptome pathology will be a common feature of autism, as many of the susceptibility genes encode synapse proteins and regulators of proteostasis (37). Convergence of phenotypes arising from mutations in different classes of disease genes may also occur in schizophrenia because the susceptibility genes are enriched in proteins in PSD95 supercomplexes (22–26).

Given the synapse proteome complexity and its diversity of protein types, it is very likely that mutations in most general regulatory mechanisms (transcription, translation and protein turnover) will impact on the synaptome. Human brain diseases arise from a wide range of different genomic structural alterations including mutations affecting gene regulation, protein structure, copy number, chromosomal rearrangements and so on. All of these genomic structural changes could impact on the molecular hierarchy and thereby change the synaptome architecture. Thus, synaptome mapping in models of these genetic and cell biological mechanisms will be rich areas of investigation in the future.

## The functional importance of synaptome architecture for behavior

I have described how synapse diversity and synaptome architecture will be targeted in a very wide range of diseases, and from this it should be clear that we need to understand how the synaptome is important for behavior so that we can interpret how these diseases produce their behavioral phenotypes. Central to this issue is the need to understand the functional importance of synapse diversity in behavior, which is a subject that has received very little attention and is not part of the standard literature on synapse physiology and behavior. It is well known that synapse proteome composition controls synaptic transmission and synaptic plasticity, and thus by extension, different synapse types will show different functional properties. We have developed a theory called the Synaptomic Theory that explains how synapse diversity and synaptome maps can store information (innate and learned behaviors) that can be “recalled” by patterns of nerve cell activity (38).

Because the release of a neurotransmitter generates a postsynaptic response amplitude that is modulated during the train of activity, synapses with different proteomes show different patterns of response. This means that the proteome of a synapse type can be identified by its response to a pattern of activity. In other words, the information stored in the proteome of individual synapses can be functionally accessed or recalled by examining the response of that synapse to a pattern of activity. The spatial distribution of these different synapse types on dendrites, cell types and brain regions will control the output from their relevant circuits. Normal and mutant synaptome maps produce different outputs that could drive different behaviors.

## Concluding comments and future perspectives

One of the most powerful features of synapse proteome and synaptome biology is their direct connection to the genome and hence genetic disorders. A huge number of diseases directly target the genes encoding the synapse proteome, and these could all result in altered synaptome architecture. A further set of diseases targeting regulatory proteins could also result in changes to the synaptome. It seems probable that genetic disorders that interfere with non-neuronal cells may in some cases alter the synaptome too, since astrocytes and microglia are known to modulate synapse biology. Because synapse proteomes are spatially distributed into diverse synapses and they are distributed into an architecture, the synaptome and its hierarchical molecular assembly provide a roadmap from the gene to the brain circuit and to behavior that can be applied in genetic disorders of the brain.

Synaptomic methods are in their infancy and there is a need to enhance and develop many aspects of the technology. Alongside the molecular labeling and imaging technology, there is a need to develop the nascent Mouse Synaptome Atlas resource and build other resources for the dissemination of data and integration with other brain atlases, connectomes, genomic and proteomic databases. There are no systematic synapse catalogs that embrace our current knowledge of synapse proteome complexity. Programs of research that map the synaptome will be required in human and in model organisms. We have begun to apply the SynMap pipeline (31) developed for the mouse to the human brain and found that it is possible to map synaptomes in normal and diseased human postmortem tissue. This opens the possibility of directly studying the synaptome neuropathology in human brain and comparing the species differences in synapse diversity and synaptome architecture.

The synaptome contains three-dimensional molecular information about brain structure and function and has the potential to link with the established brain imaging methods used in the clinic. We have shown that the differential synapse proteome composition of regions of the human neocortex correlates with functional brain imaging (Positron Emission Tomography (PET) and fMRI) (39) and that the mouse synaptome network topology correlates with the resting state fMRI network (31). These findings are a step toward using fMRI and PET imaging to study the synaptome in living individuals over the lifespan.

In conclusion, the complexity of the synapse proteome and the remarkable and beautiful architecture of the synaptome are a framework that enables us to understand the link between genetic disorders, the architecture of the brain and behavior. Synaptomic methods will enable a new range of basic science investigations that together with genetic and clinical imaging approaches have the potential to provide a rational and general model of the genetic basis of behavioral disorders.

## References

[1] Husi H., Ward M.A., Choudhary J.S., Blackstock W.P. and Grant S.G. (2000) Proteomic analysis of NMDA receptor-adhesion protein signaling complexes. Nat Neurosci., 3, 661–669. 10862698 10.1038/76615

[2] Husi H. and Grant S.G. (2001) Isolation of 2000-kDa complexes of N-methyl-D-aspartate receptor and postsynaptic density 95 from mouse brain. J. Neurochem., 77, 281–291. 11279284 10.1046/j.1471-4159.2001.t01-1-00248.x

[3] Bayes A., Collins M.O., Croning M.D., van de Lagemaat L.N., Choudhary J.S. and Grant S.G. (2012) Comparative study of human and mouse postsynaptic proteomes finds high compositional conservation and abundance differences for key synaptic proteins. PLoS One, 7, e46683. 23071613 10.1371/journal.pone.0046683 PMC3465276

[4] Bayes A., Collins M.O., Reig-Viader R., Gou G., Goulding D., Izquierdo A., Choudhary J.S., Emes R.D. and Grant S.G. (2017) Zebrafish synapse proteome complexity, evolution and ultrastructure. Nat. Commun., 8, 14613. 28252024 10.1038/ncomms14613 PMC5337974

[5] Bayes A., van de Lagemaat L.N., Collins M.O., Croning M.D., Whittle I.R., Choudhary J.S. and Grant S.G. (2011) Characterization of the proteome, diseases and evolution of the human postsynaptic density. Nat. Neurosci., 14, 19–21. 21170055 10.1038/nn.2719 PMC3040565

[6] Collins M.O., Husi H., Yu L., Brandon J.M., Anderson C.N., Blackstock W.P., Choudhary J.S. and Grant S.G. (2006) Molecular characterization and comparison of the components and multiprotein complexes in the postsynaptic proteome. J. Neurochem., 97(Suppl 1), 16–23. 16635246 10.1111/j.1471-4159.2005.03507.x

[7] Collins M.O., Yu L., Coba M.P., Husi H., Campuzano I., Blackstock W.P., Choudhary J.S. and Grant S.G. (2005) Proteomic analysis of in vivo phosphorylated synaptic proteins. J. Biol. Chem., 280, 5972–5982. 15572359 10.1074/jbc.M411220200

[8] Distler U., Schmeisser M.J., Pelosi A., Reim D., Kuharev J., Weiczner R., Baumgart J., Boeckers T.M., Nitsch R., Vogt J. et al. (2014) In-depth protein profiling of the postsynaptic density from mouse hippocampus using data-independent acquisition proteomics. Proteomics, 14, 2607–2613. 25211037 10.1002/pmic.201300520

[9] Emes R.D., Pocklington A.J., Anderson C.N., Bayes A., Collins M.O., Vickers C.A., Croning M.D., Malik B.R., Choudhary J.S., Armstrong J.D. et al. (2008) Evolutionary expansion and anatomical specialization of synapse proteome complexity. Nat. Neurosci., 11, 799–806. 18536710 10.1038/nn.2135 PMC3624047

[10] Peng J., Kim M.J., Cheng D., Duong D.M., Gygi S.P. and Sheng M. (2004) Semiquantitative proteomic analysis of rat forebrain postsynaptic density fractions by mass spectrometry. J. Biol. Chem., 279, 21003–21011. 15020595 10.1074/jbc.M400103200

[11] Lisman J. (1994) The CaM kinase II hypothesis for the storage of synaptic memory. Trends Neurosci., 17, 406–412. 7530878 10.1016/0166-2236(94)90014-0

[12] Komiyama N.H., van de Lagemaat L.N., Stanford L.E., Pettit C., Strathdee D.J., Strathdee J.E., Fricker D.G., Tuck E.J., Elsegood K.A., Ryan T.J. et al. (2018) Synaptic combinatorial molecular mechanisms generate repertoires of innate and learned behavior. 10.1101/500389

[13] Kopanitsa M.V., van de Lagemaat L.N., Afinowi N.O., Strathdee D.J., Strathdee K.E., Fricker D.G., Tuck E.J., Elsegood K.A., Croning M.D., Komiyama N.H. et al. (2018) A combinatorial postsynaptic molecular mechanism converts patterns of nerve impulses into the behavioral repertoire. 10.1101/500447

[14] Coba M.P., Pocklington A.J., Collins M.O., Kopanitsa M.V., Uren R.T., Swamy S., Croning M.D., Choudhary J.S. and Grant S.G. (2009) Neurotransmitters drive combinatorial multistate postsynaptic density networks. Sci Signal, 2, ra19. 19401593 10.1126/scisignal.2000102 PMC3280897

[15] Pocklington A.J., Cumiskey M., Armstrong J.D. and Grant S.G. (2006) The proteomes of neurotransmitter receptor complexes form modular networks with distributed functionality underlying plasticity and behaviour. Mol Syst Biol, 2, 2006.0023. 10.1038/msb4100041 PMC1681474 16738568

[16] Ajay S.M. and Bhalla U.S. (2006) Synaptic plasticity in vitro and in silico: insights into an intracellular signaling maze. Physiology (Bethesda), 21, 289–296. 16868318 10.1152/physiol.00009.2006

[17] Gavin A.C., Aloy P., Grandi P., Krause R., Boesche M., Marzioch M., Rau C., Jensen L.J., Bastuck S., Dumpelfeld B. et al. (2006) Proteome survey reveals modularity of the yeast cell machinery. Nature, 440, 631–636. 16429126 10.1038/nature04532

[18] Krogan N.J., Cagney G., Yu H., Zhong G., Guo X., Ignatchenko A., Li J., Pu S., Datta N., Tikuisis A.P. et al. (2006) Global landscape of protein complexes in the yeast Saccharomyces cerevisiae. Nature, 440, 637–643. 16554755 10.1038/nature04670

[19] Frank R.A., Komiyama N.H., Ryan T.J., Zhu F., O'Dell T.J. and Grant S.G. (2016) NMDA receptors are selectively partitioned into complexes and supercomplexes during synapse maturation. Nat. Commun., 7, 11264. 27117477 10.1038/ncomms11264 PMC5227094

[20] Komiyama N.H., Watabe A.M., Carlisle H.J., Porter K., Charlesworth P., Monti J., Strathdee D.J., O'Carroll C.M., Martin S.J., Morris R.G. et al. (2002) SynGAP regulates ERK/MAPK signaling, synaptic plasticity, and learning in the complex with postsynaptic density 95 and NMDA receptor. J. Neurosci., 22, 9721–9732. 12427827 10.1523/JNEUROSCI.22-22-09721.2002 PMC6757832

[21] Migaud M., Charlesworth P., Dempster M., Webster L.C., Watabe A.M., Makhinson M., He Y., Ramsay M.F., Morris R.G., Morrison J.H. et al. (1998) Enhanced long-term potentiation and impaired learning in mice with mutant postsynaptic density-95 protein. Nature, 396, 433–439. 9853749 10.1038/24790

[22] Fernandez E., Collins M.O., Frank R.A.W., Zhu F., Kopanitsa M.V., Nithianantharajah J., Lempriere S.A., Fricker D., Elsegood K.A., McLaughlin C.L. et al. (2017) Arc requires PSD95 for assembly into postsynaptic complexes involved with neural dysfunction and intelligence. Cell Rep., 21, 679–691. 29045836 10.1016/j.celrep.2017.09.045 PMC5656750

[23] Fernandez E., Collins M.O., Uren R.T., Kopanitsa M.V., Komiyama N.H., Croning M.D., Zografos L., Armstrong J.D., Choudhary J.S. and Grant S.G. (2009) Targeted tandem affinity purification of PSD-95 recovers core postsynaptic complexes and schizophrenia susceptibility proteins. Mol. Syst. Biol., 5, 269. 19455133 10.1038/msb.2009.27 PMC2694677

[24] Fromer M., Pocklington A.J., Kavanagh D.H., Williams H.J., Dwyer S., Gormley P., Georgieva L., Rees E., Palta P., Ruderfer D.M. et al. (2014) De novo mutations in schizophrenia implicate synaptic networks. Nature, 506, 179–184. 24463507 10.1038/nature12929 PMC4237002

[25] Kirov G., Pocklington A.J., Holmans P., Ivanov D., Ikeda M., Ruderfer D., Moran J., Chambert K., Toncheva D., Georgieva L. et al. (2012) De novo CNV analysis implicates specific abnormalities of postsynaptic signalling complexes in the pathogenesis of schizophrenia. Mol. Psychiatry, 17, 142–153. 22083728 10.1038/mp.2011.154 PMC3603134

[26] Purcell S.M., Moran J.L., Fromer M., Ruderfer D., Solovieff N., Roussos P., O’Dushlaine C., Chambert K., Bergen S.E., Kahler A. et al. (2014) A polygenic burden of rare disruptive mutations in schizophrenia. Nature, 506, 185–190. 24463508 10.1038/nature12975 PMC4136494

[27] Schreiner D., Nguyen T.M., Russo G., Heber S., Patrignani A., Ahrne E. and Scheiffele P. (2014) Targeted combinatorial alternative splicing generates brain region-specific repertoires of neurexins. Neuron, 84, 386–398. 25284007 10.1016/j.neuron.2014.09.011

[28] O'Rourke N.A., Weiler N.C., Micheva K.D. and Smith S.J. (2012) Deep molecular diversity of mammalian synapses: why it matters and how to measure it. Nat. Rev. Neurosci., 13, 365–379. 22573027 10.1038/nrn3170 PMC3670986

[29] Zhu F., Collins M.O., Harmse J., Choudhary J.S., Grant S.G. and Komiyama N.H. (2018) Cell-type specific visualization and biochemical isolation of endogenous synaptic proteins in mice. *bioRxiv*, in press. 10.1111/ejn.14597 PMC7079123 31621109

[30] Grant S.G. (2007) Toward a molecular catalogue of synapses. Brain Res. Rev., 55, 445–449. 17572504 10.1016/j.brainresrev.2007.05.003

[31] Zhu F., Cizeron M., Qiu Z., Benavides-Piccione R., Kopanitsa M.V., Skene N.G., Koniaris B., DeFelipe J., Fransen E., Komiyama N.H. et al. (2018) Architecture of the mouse brain synaptome. Neuron, 99, 781–799 e710. 30078578 10.1016/j.neuron.2018.07.007 PMC6117470

[32] DeFelipe J. (2010) From the connectome to the synaptome: an epic love story. Science, 330, 1198–1201. 21109663 10.1126/science.1193378

[33] Skene N.G., Roy M. and Grant S.G. (2017) A genomic lifespan program that reorganises the young adult brain is targeted in schizophrenia. Elife, 6. 10.7554/eLife.17915 PMC5595438 28893375

[34] Frank R.A. and Grant S.G. (2017) Supramolecular organization of NMDA receptors and the postsynaptic density. Curr. Opin. Neurobiol., 45, 139–147. 28577431 10.1016/j.conb.2017.05.019 PMC5557338

[35] Frank R.A.W., Zhu F., Komiyama N.H. and Grant S.G.N. (2017) Hierarchical organization and genetically separable subfamilies of PSD95 postsynaptic supercomplexes. J. Neurochem., 142, 504–511. 28452394 10.1111/jnc.14056 PMC5601282

[36] Wang G.X., Smith S.J. and Mourrain P. (2014) Fmr1 KO and fenobam treatment differentially impact distinct synapse populations of mouse neocortex. Neuron, 84, 1273–1286. 25521380 10.1016/j.neuron.2014.11.016 PMC4479348

[37] Louros S.R. and Osterweil E.K. (2016) Perturbed proteostasis in autism spectrum disorders. J. Neurochem., 139, 1081–1092. 27365114 10.1111/jnc.13723 PMC5215415

[38] Grant S.G.N. (2019) The synaptomic theory of behavior and brain disease. Cold Spring Harb. Symp. Quant. Biol., doi: 10.1101/sqb.2018.83.037887. 30886054

[39] Roy M., Sorokina O., Skene N., Simonnet C., Mazzo F., Zwart R., Sher E., Smith C., Armstrong J.D. and Grant S.G.N. (2018) Proteomic analysis of postsynaptic proteins in regions of the human neocortex. Nat. Neurosci., 21, 130–138. 29203896 10.1038/s41593-017-0025-9

